# Characterization of the Complete Nucleotide Sequences of *mcr-1*-Encoding Plasmids From Enterobacterales Isolates in Retailed Raw Meat Products From the Czech Republic

**DOI:** 10.3389/fmicb.2020.604067

**Published:** 2021-01-15

**Authors:** Marketa Zelendova, Costas C. Papagiannitsis, Adam Valcek, Matej Medvecky, Ibrahim Bitar, Jaroslav Hrabak, Tereza Gelbicova, Alzbeta Barakova, Iva Kutilova, Renata Karpiskova, Monika Dolejska

**Affiliations:** ^1^Central European Institute of Technology, University of Veterinary and Pharmaceutical Sciences Brno, Brno, Czechia; ^2^Department of Biology and Wildlife Diseases, Faculty of Veterinary Hygiene and Ecology, University of Veterinary and Pharmaceutical Sciences Brno, Brno, Czechia; ^3^Department of Microbiology, Faculty of Medicine and University Hospital in Plzen, Charles University, Plzen, Czechia; ^4^Faculty of Medicine, Biomedical Center, Charles University, Plzen, Czechia; ^5^Department of Microbiology, University Hospital of Larissa, Larissa, Greece; ^6^Department of Bacteriology, Veterinary Research Institute, Brno, Czechia; ^7^Department of Experimental Biology, Faculty of Science, Masaryk University Brno, Brno, Czechia

**Keywords:** antimicrobial resistance, colistin, IncHI2, IncI2, IncX4, meat

## Abstract

The aim of our study was to determine complete nucleotide sequence of *mcr-1-*carrying plasmids from Enterobacterales isolates recovered from domestic and imported raw retailed meat and compare them with plasmids available at the GenBank sequence database. A set of 16 plasmids originating from *Escherichia coli* (*n* = 13), *Klebsiella pneumoniae* (*n* = 2), and *Citrobacter braakii* (*n* = 1) were analyzed. In our previous study, data from whole genome sequencing showed that *mcr-1* gene was located on plasmids of different incompatibility groups (IncHI2, IncI2, and IncX4). The IncI2 (*n* = 3) and IncX4 (*n* = 8) plasmids harbored *mcr-1.1* gene only, whereas IncHI2 sequence type 4 plasmids (*n* = 5) carried large multidrug resistance (MDR) regions. MDR regions of IncHI2 plasmids included additional antimicrobial resistance genes conferring resistance to β-lactams (*bla*_TEM−1_), aminoglycosides [*aadA1, aadA2*, and *aph(6)-Id*], macrolides [*mef* (B)], tetracycline (*tetA, tetR*), and sulphonamides (*sul1, sul2*, and *sul3*). Likewise, IncHI2 plasmids carried several insertion sequences including IS*1*, IS*3*, IS*26*, IS*1326*, and IS*Apl1*. In conclusion, our findings confirmed the involvement of IncX4, IncI2, and IncHI2 plasmids in the dissemination of *mcr-1.1* gene in several environmental niches, as in samples of retail meat originating from different geographical regions. In contrast to IncX4 and IncI2, IncHI2 plasmids were more diverse and carried additional genes for resistance to heavy metals and multiple antimicrobials.

## Introduction

The emergence of new genetic elements encoding antimicrobial resistance in bacterial pathogens represents a threat to public health. Polymyxins are cationic polypeptide antimicrobials and include five different compounds (polymyxin A–E) of which only two compounds, polymyxin B and polymyxin E (colistin), are used clinically (Falagas et al., 2010). Colistin is considered as a last resort antimicrobial agent against multi-drug resistant Gram-negative bacteria including Enterobacterales. However, the occurrence and spread of colistin-resistant bacteria has rapidly increased worldwide (Li et al., 2017).

Resistance to colistin can be mediated via chromosomal mutations in genes involved in lipopolysaccharide synthesis (Olaitan et al., 2014). These mutations mediate modifications of the bacterial outer membrane through alteration of the lipopolysaccharide (Landman et al., 2008). In 2016, the first plasmid-mediated colistin resistance gene, *mcr-1*, was identified among Chinese Enterobacterales isolates (Liu et al., 2016). Since the first discovery of *mcr-1* gene in 2016 in China 10 variants of *mcr* genes have been reported (Wang et al., 2020). These genes encode phosphoethanolamine transferases that catalyze the addition of phosphoethanolamine to the phosphate group of lipid A, reducing the negative charge of the bacterial outer membrane and attenuating its affinity for colistin, resulting in antimicrobial resistance (Poirel et al., 2017).

Antimicrobial resistance genes are mostly located on conjugative plasmids that allow their efficient dissemination among bacteria. Resistance plasmids belong to diverse incompatibility groups and they usually carry a wide variety of genes conferring resistance to β-lactams, aminoglycosides, co-trimoxazole, quinolones, and other antimicrobials (Carattoli et al., 2014; Rozwandowicz et al., 2018). In Enterobacterales several major plasmid families, mainly carrying C, HI2, I1, I2, M, N, X, F, and X replicons have been found in association with emerging antimicrobial resistance determinants (Carattoli, 2013; Matamoros et al., 2017). Plasmids carrying *mcr-1* gene have been reported to include mainly IncHI2, IncI2, IncFII, IncP, and IncX4 families (Doumith et al., 2016; Xavier et al., 2016). A recent study, focused on mobile genetic elements (MGEs) carrying *mcr-1* gene, reported the regional spread of IncHI2 plasmids in Europe and of IncI2 plasmids in Asia (Matamoros et al., 2017). Another study has highlighted chicken meat as an emerging reservoir of colistin-resistant *E. coli* strains, carrying *mcr-1* on IncX4 plasmids, in South America (Monte et al., 2017). IS*Apl1* found upstream of *mcr-1* gene has been proposed to be the key element mediating translocation of *mcr-1* into various plasmid backbones (Sun et al., 2017).

In a previous study from our group, a high prevalence of Enterobacterales with *mcr*-mediated colistin resistance was observed in retail meat of different origins, including imported meat and meat from domestic production (Gelbíčová et al., 2019). Thus, the aim of the current study was to characterize the complete nucleotide sequence of *mcr-1*-carrying plasmids, which were assigned to different Inc groups, in order to examine the nature of the MGEs involved in the acquisition and spread of *mcr-1* in foodborne Enterobacterales in the Czech Republic.

## Materials and Methods

### Bacterial Isolates and Plasmids

In a previous study, a total of 61 MCR-1-producing Enterobacterales isolates were identified from meat at retail markets in the Czech Republic (Gelbíčová et al., 2019). These isolates included *E. coli* (*n* = 54), *Klebsiella pneumoniae* (*n* = 6), and *Citrobacter braakii* (*n* = 1) and were assigned to 34 different sequence types (STs), of which *E. coli* isolates belonging to ST10, ST93, and ST744 were the most common. In our follow-up study, 16 *mcr-1*-positive plasmids being representatives of IncHI2 (*n* = 5), IncI2 (*n* = 3), and IncX4 (*n* = 8) groups from isolates of different country (Brazil, Czech Republic, China, Germany and Poland) and species origin (turkey and rabbit) were selected for characterization of complete nucleotide sequences (Table 1).

**Table 1:** The characteristics and origin of *mcr-1* encoding plasmids.

**Plasmid**	**Organism**	**ST**	**Colistin MICs^a^ (mg/L)**	**Origin^b^**	***mcr-1*-encoding plasmids (bp)^c^**	**WGS data (technology)^d^**
pMCR_1139_A1	*E. coli*	ST10	8	Raw turkey meat, PL	IncX4 (33,303)	WS (Illumina)
pMCR_1138_D1	*E. coli*	ST744	8	Raw turkey meat, DE	IncX4 (33,308)	WS (Illumina)
pMCR_1253_A2	*E. coli*	ST10	8	Raw turkey meat, DE	IncX4 (33,310)	WS (Illumina)
pMCR_1449_C1	*E. coli*	ST1079	4	Raw turkey liver, BR	IncX4 (33,304)	WS (Illumina)
pMCR_1413_E1	*E. coli*	ST354	4	Raw turkey meat, CZ	IncX4 (33,304)	WS (Illumina)
pMCR_1525_B1	*K. pneumoniae*	ST147	8	Raw turkey liver, BR	IncX4 (33,304)	WS (Illumina)
pMCR_1525_C2	*K. pneumoniae*	ST11	8	Raw turkey liver, BR	IncX4 (34,081)	TC (Illumina)
pMCR_1525_D1	*E. coli*	ST349	4	Raw turkey meat, BR	IncX4 (33,300)	WS (Illumina)
pMCR_1138_A1	*E. coli*	ST162	4	Raw turkey meat, DE	IncI2-X4 (95,202)	WS (Illumina)
pMCR_1884_B3	*E. coli*	ST1196	4	Raw rabbit meat, CHN	IncI2 (60,643)	WS (Illumina)
pMCR_1884_C3	*C. braakii*	Unknown	4	Raw rabbit meat, CHN	IncI2 (60,164)	WS (Illumina)
pMCR_170_D1	*E. coli*	ST7973	4	Raw turkey meat, PL	IncHI2/ST4 (284,175)	TC (PacBio)
pMCR_915_C1	*E. coli*	ST224	4	Raw turkey meat, PL	IncHI2/ST4 (253,135)	TC (PacBio)
pMCR_915_E1	*E. coli*	ST1140	4	Raw turkey meat, PL	IncHI2/ST4 (250,657)	TC (PacBio)
pMCR_1085_C1	*E. coli*	ST756	8	Raw turkey meat, PL	IncHI2/ST4 (251,547)	TC (PacBio)
pMCR_1139_D1	*E. coli*	ST1167	4	Raw turkey meat, PL	IncHI2/ST4 (267,374)	TC (PacBio)

a *The minimum inhibitory concentration (MIC) of wildtype strains*.

b *BR, Brazil; CZ, Czech Republic; CHN, China; DE, Germany; PL, Poland*.

c *Plasmids IncHI2 were assigned to ST by plasmid multi-locus sequence typing (pMLST)*.

d *TC, transconjugant; WS, wildtype strains; plasmids from WS were retrieved from WGS data published in Gelbíčová et al. (2019) were subjected to bioinformatics analysis, PCR-based gap closing, annotation and comparative analysis*.

### Plasmid Assembly and Analysis

For isolates, harboring *mcr-1*-carrying plasmids that belonged to IncX4 or IncI2 groups, plasmid sequences were extracted from Illumina data obtained previously (Gelbíčová et al., 2019). The sequence gaps were filled by a PCR-based strategy and Sanger sequencing.

IncHI2 plasmids carrying *mcr-1* were sequenced using PacBio technology and assembled by the Hierarchical Genome Assembly Process (HGAP) v.4 (Chin et al., 2013) which provides long reads of single-molecule DNA and enables the closing of the whole plasmid sequences.

For sequence analysis and annotation, the BLAST algorithm (www.ncbi.nlm.nih.gov/BLAST), the ISfinder database (www-is.biotoul.fr/), and the ORF finder tool (www.bioinformatics.org/sms/) were utilized. The CGE online tools (https://cge.cbs.dtu.dk/) were used to identify antimicrobial resistance genes (ResFinder 4.1) (Zankari et al., 2012), plasmid replicons (PlasmidFinder 2.1), and plasmid STs (pMLST 2.0) (Carattoli et al., 2014). The Integrall integron database (http://integrall.bio.ua.pt) was used to analyze and assign integron sequences (Moura et al., 2009). Alignments with highly similar complete plasmid sequences available in NCBI were conducted using the BRIG tool (v0.95).

### Nucleotide sequence Accession Numbers

The sequences of our reported plasmids have been deposited in GenBank under accession numbers MT929275, MT929276, MT929277, MT929278, MT929279, MT929280, MT929281, MT929282, MT929283, MT929284, MT929285, MT929286, MT929287, MT929288, MT929289, and MT929290.

## Results

### Characteristics of *mcr-1*-Carrying Plasmids

Sixteen *mcr-1*-positive plasmids, selected for this study, included three main groups: (i) two ~60 kb IncI2 plasmids originated from raw rabbit meat imported from China, (ii) five IncHI2 plasmids with size between ~250 and 290 kb originated from raw turkey meat imported from Poland, and (iii) IncX4 group (*n* = 8) which sized ~33 kb and predominated our collection (Table 1). The IncI2-X4 hybrid plasmid (pMCR_1138_A1) of ~95 kb was obtained from raw turkey meat imported from Germany. The *mcr-1.1* genes from all wild strains were transferred to sodium azide-resistant *E. coli* J53 K12 laboratory strain by conjugation (Gelbíčová et al., 2019), confirming the ability of *mcr-1.1*-carrying plasmids to further disseminate the *mcr-1.1* in other clones or species.

Fifteen of the plasmids carried a complete *mcr-1.1* gene, whereas one IncHI2 plasmid (pMCR_1139_D1) carried a *mcr-1.1* gene with a truncation of the 3′ end, which was bordered by two IS*Apl1* elements. Plasmids belonging to IncI2 and IncX4 groups (including the hybrid strain), did not carry additional antimicrobial resistance genes, whereas IncHI2 plasmids exhibited large MDR regions.

### Structure of IncX4 *mcr-1.1*-Carrying Plasmids

**Figure 1:**
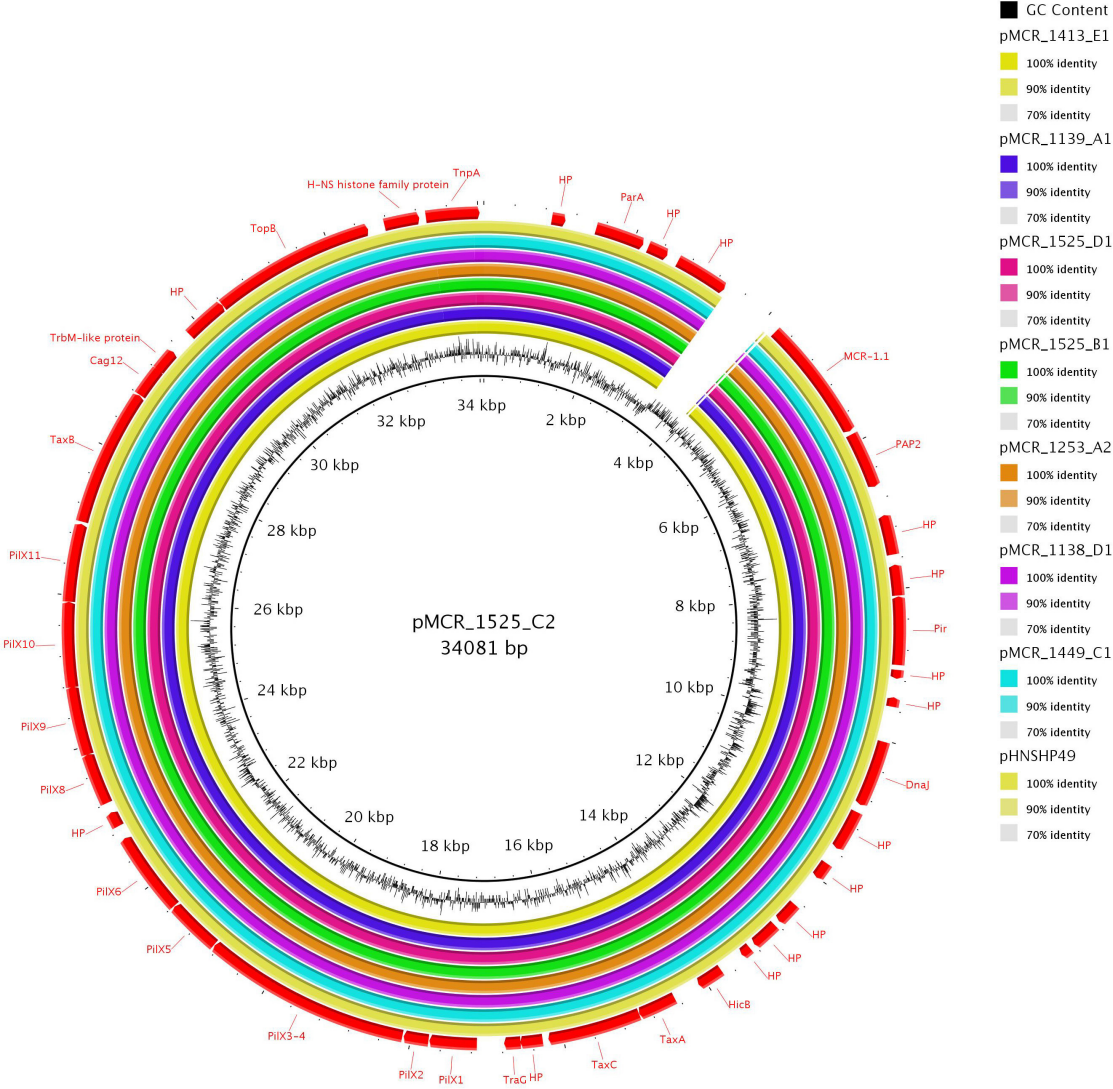
Sequence alignment of eight IncX4 and one hybrid IncX4-I2 *mcr-1*-encoding plasmids. The plasmid pMCR_1525_C2 (GenBank accession no. MT929281) was used as a reference for the comparison.

IncX4 plasmids carrying *mcr-1.1* originated from *E. coli* and *K. pneumoniae* isolates recovered from raw turkey meat or liver from Brazil, Germany, Poland and the Czech Republic (Table 1). They were all derivatives of the plasmid pHNSHP49 that was described in the *E. coli* strain SHP49 recovered from a pig in China (GenBank accession no. MF774188). Seven out of the eight sequenced IncX4 plasmids showed high degrees of similarity to each other and to pHNSHP49 (100% coverage, 99% identity) (Figure 1). The *mcr-1.1* gene was bordered by two ORFs encoding a hypothetical protein and a PAP2 transmembrane protein. Plasmid pMCR_1525_C2, which was present in *K. pneumoniae* ST11 isolate originating from Brazil, differed from pHNSHP49 by the insertion of IS*1A* element, upstream of *mcr-1.1* gene.

### Structure of IncI2 *mcr-1.1*-Carrying Plasmids

**Figure 2:**
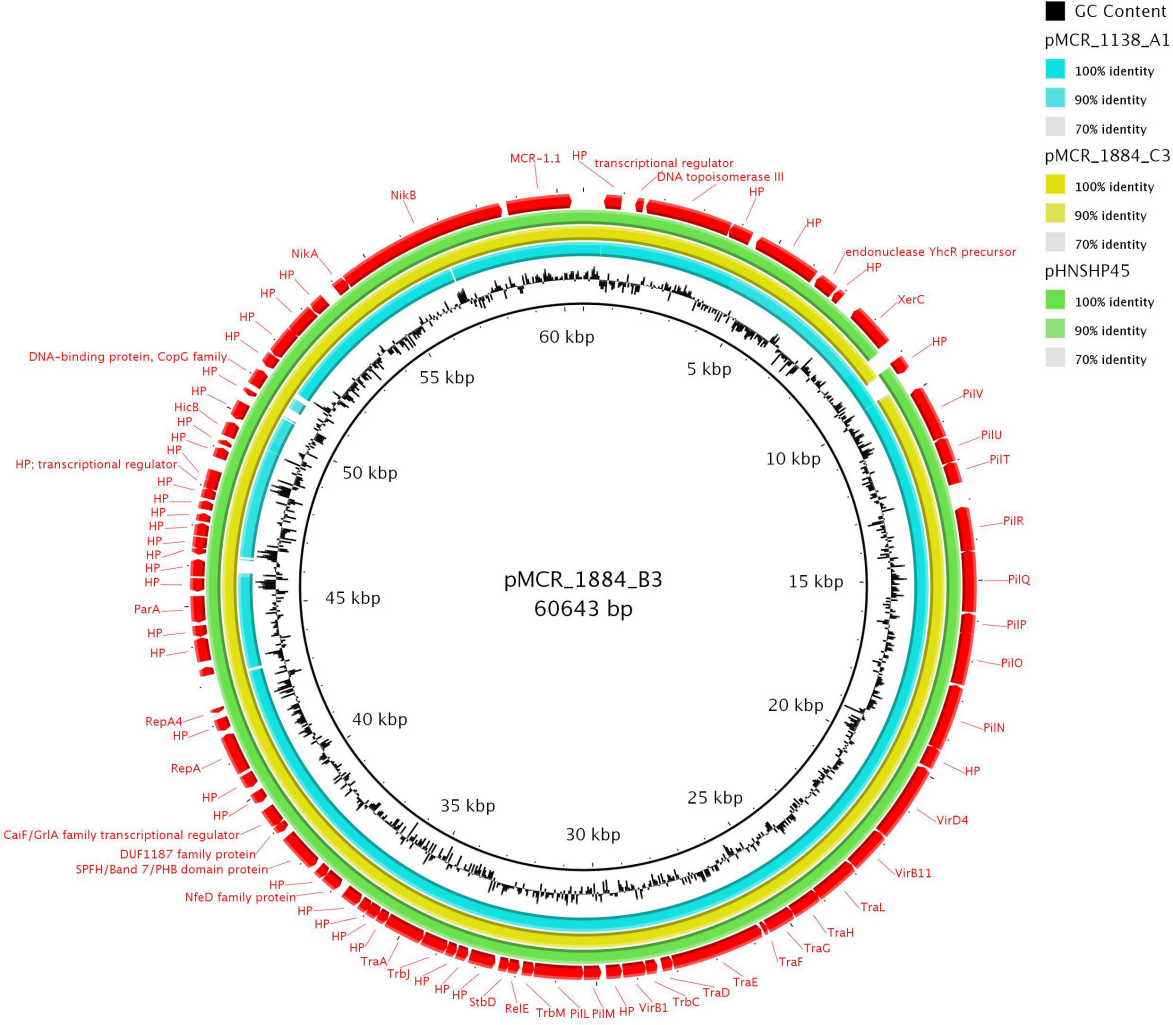
Sequence alignment of two IncI2 and one hybrid IncX4-I2 *mcr-1*-encoding plasmids. The plasmid pMCR_1884_B3 (GenBank accession no. MT929283) was used as reference to compare with other plasmids.

Two IncI2 plasmids (pMCR_1884_B3 and pMCR_1884_C3) shared high sequence identity with *mcr-1.1*-positive plasmid pHNSHP45 (99% coverage, 99% identity) (GenBank accession number KP347127) which was isolated from swine in China (Liu et al., 2016) (Figure 2). The *mcr-1.1* region was inserted downstream the *nikB* gene of IncI2 plasmid backbone, as found in pHNSHP45. Unlike pHNSHP45, an IS*Apl1* element was not found upstream *mcr-1.1* gene.

Furthermore, plasmid pMCR_1138_A1, being an IncI2-X4 hybrid, was characterized from an *E. coli* ST162 isolate originating from Germany. Sequencing data suggested that the progenitor of pMCR_1138_A1 is an IncX4 plasmid, carrying *mcr-1.1*. At a certain step of the evolution of pMCR_1138_A1, an IncX4 plasmid, carrying *mcr-1.1*, and an IncI2 plasmid may have formed a fusion structure. Fusion structure may have been formed *via* a recombination event between a homologous region (nt 29,159–31,608 in pMCR_1525_D1; Supplementary Figure 1) encoding a DNA topoisomerase III. Recombination event resulted in duplication of homologous region, in pMCR_1138_A1. Additionally, pMCR_1138_A1 harbored a Tn*3*-like transposon, which carried *bla*_TEM−32_ gene. The Tn*3*-like structure was found upstream IncX4 *parA* gene.

### Structure of *mcr-1*-Encoding IncHI2 Plasmids

**Figure 3:**
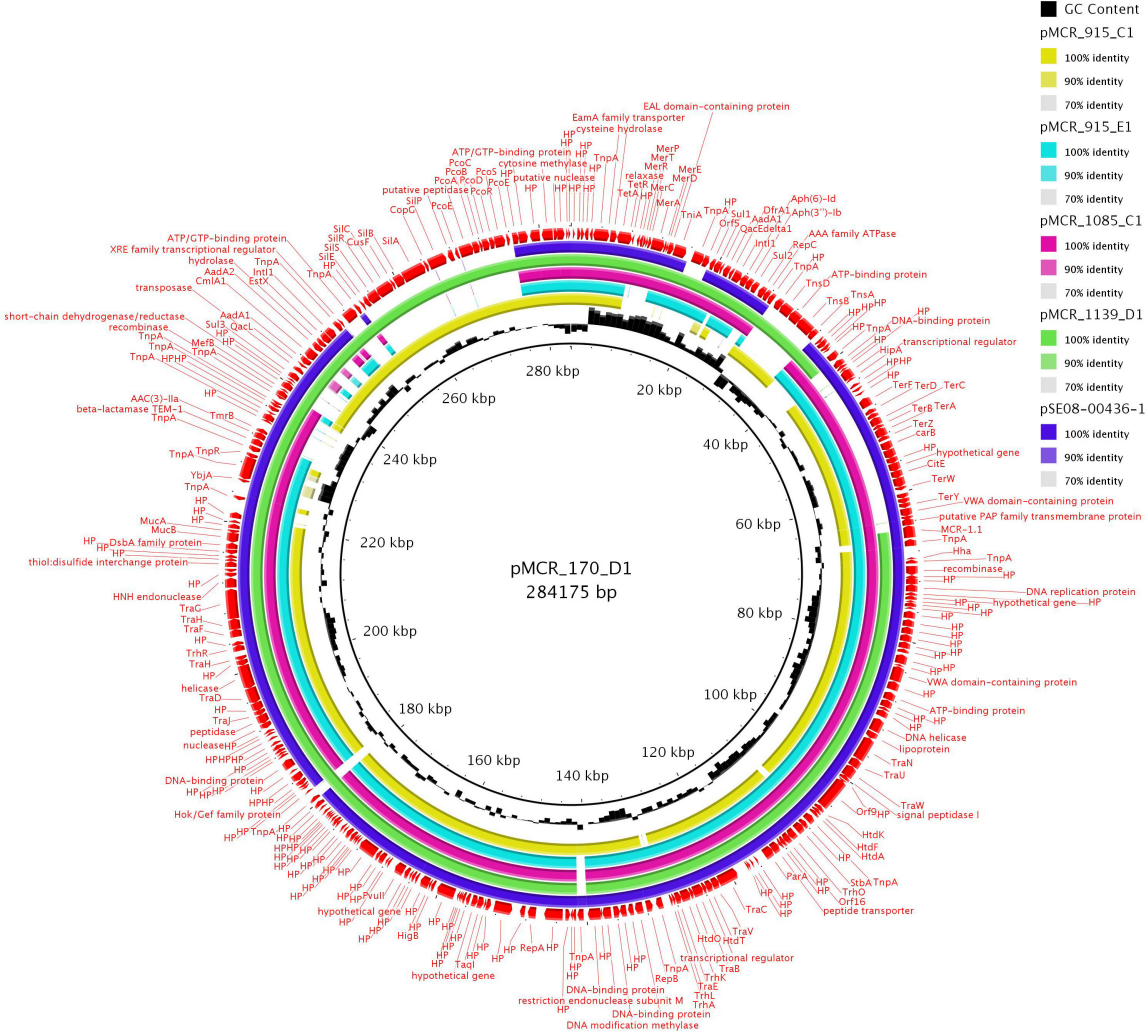
Circular representation of IncHI2 *mcr-1*-encoding plasmids. Plasmid pMCR_170_D1 (GenBank accession no. MT929288) was used as a reference for the comparison.

All five IncHI2 plasmids exhibited sequences closely related to other *mcr-1-*carrying IncHI2 plasmids, like pSE08-00436-1 from a *Salmonella enterica* strain recovered from a chicken (GenBank accession no. CP020493) in Germany (Figure 3) and belonged to ST4. IncHI2 plasmid backbones were composed of regions for replication (*reHI2*), conjugative transfer (*tra* genes), and plasmid maintenance (*par* gene). Additionally, IncHI2 plasmids (except pMCR_1139_D1) carried tellurium resistance genes (*terZABCDEF*), commonly associated with this plasmid family, in addition to *terY1, terY2*, and *terW* (Zingali et al., 2020). Also, IncHI2 plasmids (except pMCR_915_E1 and pMCR_1085_C1) carried the operons encoding *sil* and *pco*, conferring resistance to copper and silver. In all IncHI2 plasmids, *mcr-1.1* gene was inserted downstream the *terY2* gene, as observed in other IncHI2 plasmids like pSE08-00436-1 (GenBank accession no. CP020493) and pEGY1-MCR-1 (GenBank accession no. CP023143). In plasmids pMCR_170_D1, pMCR_915_E1, and pMCR_1085_C1, *mcr-1.1* gene was bounded by an IS*Apl1* element and an ORF encoding PAP2 transmembrane protein while in pMCR_915_C1, an IS*1* was found upstream *mcr-1.1* gene. Deletion of the second copy of IS*Apl1* element, flanking the *mcr-1.1* segment, was observed in the IncHI2 plasmids mentioned above. As mentioned above, in IncHI2 plasmid pMCR_1139_D1, *mcr-1.1* gene was truncated at the 3′ end due to insertion of a second IS*Apl1* element. Insertion of IS*Apl1* also deleted the ORF encoding PAP2 transmembrane protein and the adjacent *ter* operon.

**Figure 4:**
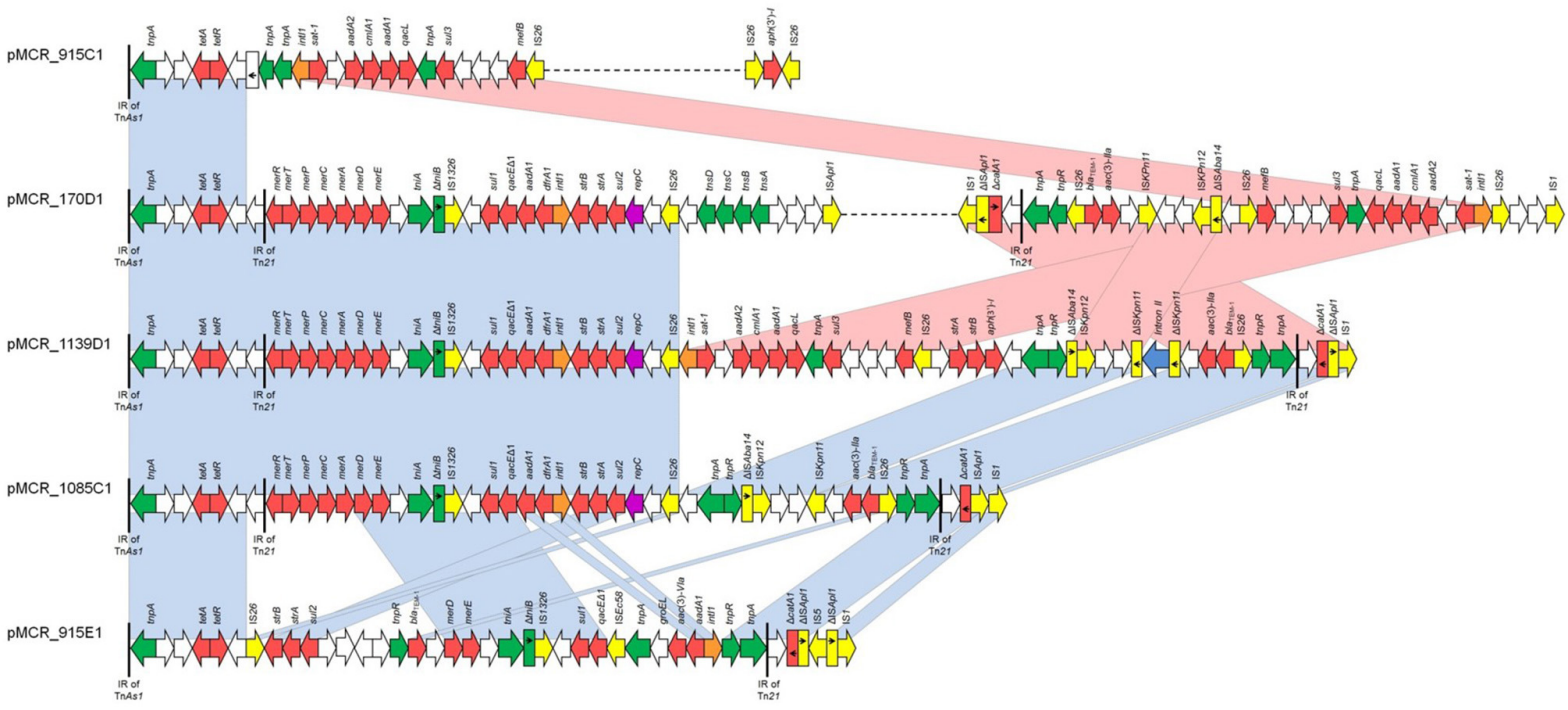
Linear maps of the multidrug resistance regions of IncHI2 plasmids, carrying *mcr-1.1* genes. Arrows show the direction of transcription of open reading frames (ORFs), while truncated ORFs appear as rectangles (arrows within rectangles indicate the direction of transcription). Antimicrobial resistance genes are shown in red. IS elements and transposases are shown in yellow and green, respectively. *intI1* genes are shaded orange. The remaining genes are shown in white. Homologous segments (representing ≥99% sequence identity) are indicated by light blue shading, while pink shading shows inverted homologous segments.

Additionally, at least one MDR region was identified in each IncHI2 plasmid. MDR regions ranged from 18,305 to 63,635 bp in size (Table 2). In all IncHI2 plasmids, the MDR regions were inserted in the same site, including a Tn*1721*-specific tetracycline module (Figure 4). The Tn*21*-specific mercury resistance module was found next to Tn*1721*-specific sequence in plasmids pMCR_170_D1, pMCR_1085_C1, and pMCR_1139_D1. The latter plasmids included also the class 1 integron In369, comprising *dfrA1b* and *aadA1b* cassettes, and a streptomycin resistance module, consisting of *sul2, strA*, and *strB*. The streptomycin resistance module has originated from plasmid RSF1010 (Yau et al., 2010), as indicated by IncQ-derived segment (nts 23,451–27,940 in pMCR_170_D1) containing *repAC* operon found next to it. Additionally, the *sul3*-associated class 1 integron, In641, carrying the *estX-3, psp, aadA2, cmlA1, aadA1a*, and *qacH2* cassettes, was identified in plasmids pMCR_170_D1, pMCR_915_C1, and pMCR_1139_D1. In641 was followed by *tnp440* transposase and a *sul3*-*orfB-orfA*-*mefB* module. The Tn*21* transposition module, and the *bla*_TEM−1_ and *aac(3)-IIa* resistance genes were also found in the MDR regions of pMCR_170_D1 and pMCR_1139_D1.

**Table 2:** The additional antimicrobial resistance genes and mobile genetic elements in IncHI2 plasmids.

**Plasmid**	**Additional antimicrobial resistance genes**	**Mobile genetic elements**	**MDR region (nts)**	**GenBannk accession no**.
pMCR_170_D1	*aadA1, aadA2, aac(3)-IIa, catA1, cmlA1, mefB, sul1, sul2, sul3, bla*_TEM−1_*, tetA, tetR*	IS*1*, IS*2*, IS*3*, IS*26*, IS*150*, IS*1326*, IS*Apl1*, IS*Kpn11*, IS*Kpn12*	2,876–40,138 and 223,049–253,900	MT929288
pMCR_915_C1	*aadA1, aadA2, aph(3′)-Ia, cmlA1, sul3, tetA, tetR*	IS*1*, IS*2*, IS*26*	2,876–21,180 and 206,165–209,312	MT929284
pMCR_915_E1	*aadA1, aph(3″)-Ia, aph(6)-Id, strA, sul1, sul2, bla*_TEM−1_*, tetA, tetR*	IS*1*, IS*3*, IS*26*, IS*1326*, IS*Apl1*	2,876–43,485	MT929285
pMCR_1085_C1	*aadA1, aph(3″)-Ib, aph(6)-Id, dfrA1, sul1, sul2, bla*_TEM−1_*, tetA, tetR*	IS*1*, IS*3*, IS*21*, IS*26*, IS*1326*, IS*Apl1*, IS*Kpn11*, IS*Kpn12*	2,876–47,789	MT929286
pMCR_1139_D1	*aadA1, aadA2, aac(3)-IIa, aph(3″)-Ia, aph(3″)-Ib, aph(6)-Id, dfrA1, cmlA1, mefB, sul1, sul2, sul3, bla*_TEM−1_*, tmrB, tetA, tetR*	IS*1*, IS*3*, IS*26*, IS*150*, IS*1326*, IS*Apl1*, IS*Kpn12*	2,876–66,510	MT929287

Furthermore, a composite transposon containing kanamycin-resistance gene *aphA1* bounded by two IS*26* elements in inverse orientation was identified in a distinct region of pMCR_915_C1.

On the other hand, in plasmid pMCR_915_E1, a streptomycin resistance module bounded by an IS*26*, and a module with the *bla*_TEM−1b_ gene derived from a Tn*2* transposon were found next to Tn*1721*-specific sequence. These modules have been previously described in Tn*6029*-like transposons (Chowdhury et al., 2015). Also, pMCR_915_C1 included the class 1 integron In2 inserted in the Tn*21* backbone. Finally, in all IncHI2 plasmids, several intact and defective mobile elements that could be involved in further reorganization of MDR regions were identified (Figure 4).

## Discussion

The plasmid-mediated colistin resistance in Enterobacterales has been reported worldwide (Monte et al., 2017; Tijet et al., 2017; Wang et al., 2018). In our study, 16 *mcr-1.1*-carrying plasmids, belonging to three different Inc groups (IncI2, IncX4, and IncHI2) and originating from different geographical areas were completely sequenced, closed and their comparative analysis was performed. IncI2, IncX4, and IncHI2 plasmid groups have been frequently reported in association with *mcr-1* gene (Li et al., 2017; Matamoros et al., 2017; Tijet et al., 2017), highlighting their involvement in the dissemination of this important resistance determinant. IncI2 plasmids characterized from bacteria recovered from raw rabbit meat imported from China, exhibited high sequence homology to previously characterized plasmid pHNSHP45 from China. This finding, in agreement with the data of a previous study (Matamoros et al., 2017), confirmed the origin of IncI2 plasmid, carrying *mcr-1.1* resistance gene, from China. The first described IncI2 plasmid carrying *mcr-1* gene originated from *E. coli* from swine in China (Liu et al., 2016). On the other hand, IncX4 *mcr-1.1*-carrying plasmids, which were highly similar to each other, originated from raw turkey meat and liver of different geographical areas including Czech Republic, Brazil, Poland, and Germany. The occurrence of IncX4 plasmids carrying *mcr-1* of poultry meat origin has been reported in Brazil (Moreno et al., 2019), China (Sun et al., 2017), South America (Monte et al., 2017), and Switzerland (Donà et al., 2017). These data confirmed the worldwide spread of IncX4 *mcr-1.1*-carrying plasmids, underlining the important role of IncX4 plasmid group in the dissemination of clinically significant resistance determinants. Interestingly, in most IncI2 and IncX4 plasmids carrying *mcr-1.1*, no insertion sequence (IS) that could be involved in the spread of the specific resistance gene was found. Previous reports have also described the absence of ISs in association with *mcr* genes (Caltagirone et al., 2017; Donà et al., 2017). Only, in IncX4 plasmid pMCR_1525_C2 isolated from *K. pneumoniae* ST11 originating from Brazil, an IS*1A* element was upstream of *mcr-1.1*. The presence of IS*1*-like elements upstream of *mcr* genes has been previously reported only in IncHI2 plasmids pASSD2-MCR1 (GenBank accession no. KX856065) and p2017.02.01CC (GenBank accession no. LC511657). This finding underlines the plethora of mobile genetic elements that could be involved in the spread of resistance determinants, as *mcr* genes.

Furthermore, IncHI2 plasmids were only found in raw turkey meat imported from Poland. A previous report revealed a regional spread of IncHI2 plasmids, carrying *mcr-1* gene, in Europe (Matamoros et al., 2017). In addition, IncHI2 plasmids, sequenced during this study, exhibited closely related to *mcr-1-*carrying IncHI2 plasmid pSE08-00436-1 from a *Salmonella enterica* strain recovered from a chicken (GenBank accession no. CP020493) in Germany. This finding further highlighted the important role of IncHI2 plasmid family in the dissemination of *mcr-1* resistance determinant in Europe. However, IncHI2 plasmids, carrying *mcr-1* gene, showed higher diversity than other plasmid groups. As previously described in other IncHI2 plasmids (Wyrsch et al., 2015; Zingali et al., 2020), diversity of these molecules was mainly observed in MDR regions, and could be explained by acquisition and/or loss of transposons, insertion sequences, and antimicrobial resistance genes. In addition, mobile elements play a significant role in the formation of hybrid plasmids and instances where resistance plasmids have fused are known (Mangat et al., 2017). As such, the presence of several mobile elements in IncHI2 plasmids represents a potential hotspot for the introduction of new resistance gene cargo. Additionally, differences among IncHI2 plasmids were observed in the presence/absence of regions involved in resistance to tellurite, silver and copper. These results are in line with previous data (Gilmour et al., 2004), showing that IncHI2 plasmids are highly heterologous and evolve through acquisition/loss of mobile genetic elements. Additionally, previous studies have reported that IncHI2 plasmids are characterized by the presence of different operons conferring resistance to heavy metals (Gilmour et al., 2004; Wyrsch et al., 2015; Fang et al., 2016). Heavy metals are found in disinfectants, soil fertilizers and livestock feed and are recognized as environmental pollutants. Therefore, it is common to identify bacterial populations, with genetic determinants conferring resistance to heavy metals, in the gastrointestinal flora of intensively reared farm animal species. Previous studies have suggested that the presence of *pco* and *sil* operons in IncHI2 plasmids may have arisen due to co-selection pressures afforded by the use of heavy metals in feed additives (Wyrsch et al., 2015; Zingali et al., 2020). In IncHI2 plasmids, *mcr-1.1* gene was found downstream of the mobile element IS*Apl1*. IS*Apl1* element has been previously associated with the mobilization and/or stability of *mcr-1* gene (Sun et al., 2016; Partridge et al., 2018), found in various plasmids belonging to different Inc groups. Although, plasmid pMCR_1139_D1 carried a truncated *mcr-1.1* gene the *E. coli* 1139D1 strain was phenotypically resistant to colistin (MIC = 4 mg/L).

Our study described complete nucleotide sequences of IncX4, IncI2, and IncHI2 plasmids, carrying *mcr-1.1*, from Enterobacterales isolates originating from retail meat of different geographical origins. The presence of *mcr-1.1* gene in Enterobacterales from retail meat has been previously reported in studies from different countries (Kluytmans-van den Bergh et al., 2016; Zajac et al., 2019; Hassen et al., 2020), pointing out the role of food-producing animals, and retail meat, as reservoirs of *mcr-1*-carrying bacteria. In the studies mentioned above, spread of *mcr-1.1* gene was associated with IncX4, IncI2, and IncHI2 plasmid types. These findings confirmed the involvement of IncX4, IncI2, and IncHI2 plasmids in the dissemination of *mcr-1.1* gene in several environmental niches, as in samples of retail meat originating from different geographical regions. In agreement with the previous data (Matamoros et al., 2017), our findings demonstrated the increased stability of IncX4 and IncI2 plasmids carrying *mcr-1.1* gene. Furthermore, in contrast to IncX4 and IncI2, IncHI2 plasmids were more diverse due to acquisition of transposons and antimicrobial resistance genes, conferring multidrug resistance and thus posing a public health threat. Colistin is recommended for the treatment of gastrointestinal infections caused by non-invasive *E. coli*, mainly in pigs, chickens, turkey, calves, and sheep. Thus, extensive consumption of colistin by farm animals may indicate the reason for the dissemination of *mcr-1*-carrying plasmids in turkey and rabbit meat. Therefore, the animal and food market can contribute to the worldwide spread of colistin-resistant bacteria. However, extensive studies combining antimicrobial consumption and resistance are limited. In conclusion, the spread of *mcr-1.1* gene in animal and food market is an alarming situation having public health, ecological and economical effects, justifying the need of surveillance programs on colistin-resistant bacteria in farm animals, especially in poultry, and in slaughterhouses.

## Data Availability Statement

The datasets generated for this study can be found in online repositories. The names of the repository/repositories and accession number(s) can be found in the article/Supplementary Material.

## Author Contributions

MZ performed the laboratory work, data analysis, and prepared the manuscript. CP performed the data analysis and prepared the manuscript. AV and MM performed the bioinformatic analysis of whole-genome sequencing data. IB and JH performed the PacBio sequencing. TG and RK provided the samples. AB helped with data analysis. IK helped with the laboratory work. MD supervised the project. All authors discussed the results.

## Conflict of Interest

The authors declare that the research was conducted in the absence of any commercial or financial relationships that could be construed as a potential conflict of interest.
