# Supplementary material for: Characterization of the Complete Nucleotide Sequences of mcr-1-Encoding Plasmids From Enterobacterales Isolates in Retailed Raw Meat Products From the Czech Republic
Source: Front Microbiol. 2021 Jan 15;11:604067. doi: 10.3389/fmicb.2020.604067 (PMC7843963; doi:10.3389/fmicb.2020.604067)
Supplement: Supplementary file 1 [file Presentation_1.PPTX]

## Slide 1
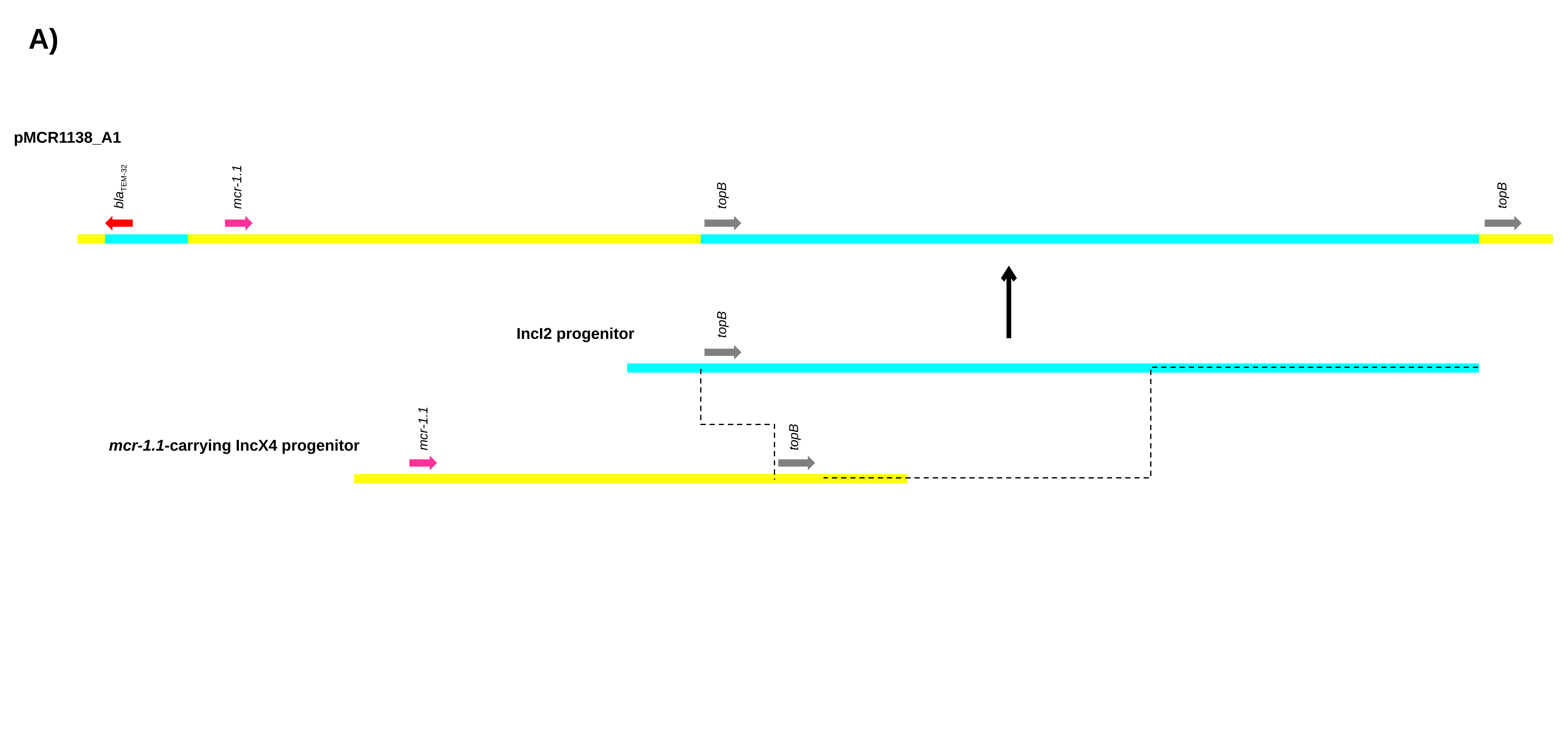

A)
pMCR1138_A1
blaTEM-32
mcr-1.1
topB
topB
topB
IncI2 progenitor
mcr-1.1
topB
mcr-1.1-carrying IncX4 progenitor

## Slide 2
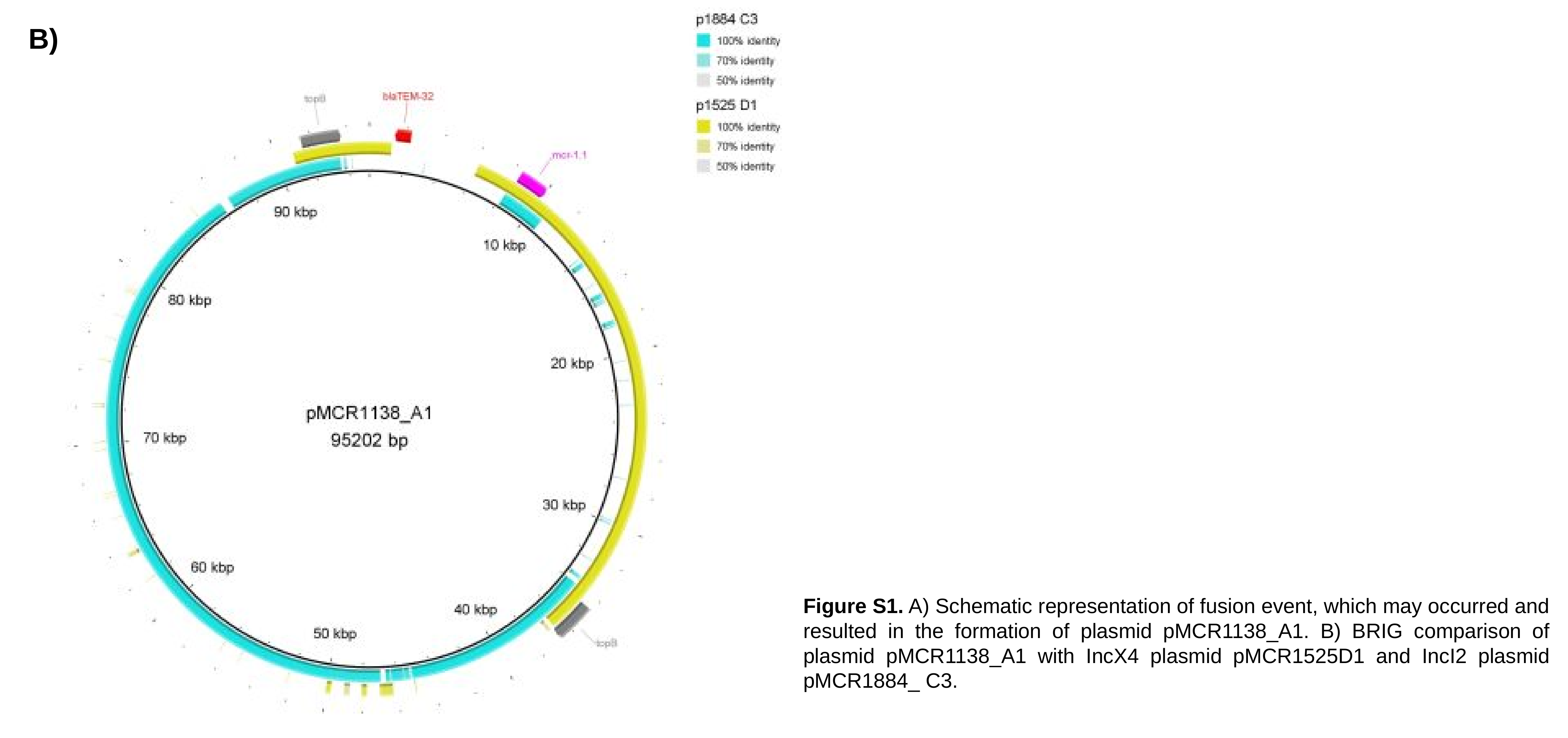

B)
Figure S1. A) Schematic representation of fusion event, which may occurred and resulted in the formation of plasmid pMCR1138_A1. B) BRIG comparison of plasmid pMCR1138_A1 with IncX4 plasmid pMCR1525D1 and IncI2 plasmid pMCR1884_ C3.
